# The role of glycemic traits in the mediation of the causal effect of hypothyroidism on coronary heart disease

**DOI:** 10.3389/fendo.2024.1436713

**Published:** 2024-09-30

**Authors:** Zhiwei Jin, Guorong Li, Zekuan Xue, Yijie Li, Wangfang Yang, Yunfei Yu, Jixue Hou

**Affiliations:** ^1^ School of Medicine, Shihezi University, Shihezi, China; ^2^ Cheeloo College of Medicine, Shandong University, Jinan, China; ^3^ Department of Thyroid and Breast Surgery, The First Affiliated Hospital of Shihezi University, Shihezi, China

**Keywords:** Mendelian randomization analysis, causality, hypothyroidism, coronary heart disease, glycemic traits

## Abstract

**Background:**

Hypothyroidism and coronary heart disease are both common diseases in life and both are increasing in prevalence. Many studies have found a strong association between the two. However, they have not been able to prove a causal relationship. Furthermore, numerous studies have demonstrated that glycemic traits play a role in both. Consequently, the objective of this study was to ascertain the causal estimation of the association between hypothyroidism and coronary heart disease and to quantify the potential mediating role of glycemic traits in this relationship.

**Methods:**

We used two-sample Mendelian randomisation (UVMR) to explore causality between hypothyroidism and coronary heart disease. Additionally, multivariate Mendelian randomisation (MVMR) was applied to quantify the potential mediation of glycemic traits in this relationship. A variety of Mendelian randomization methods were employed in this study, including the inverse variance weighting (IVW) method, weighted median method, and MR-Egger test. Heterogeneity and horizontal pleiotropy were evaluated through MR-Egger intercept test, Cochran’s Q test, and leave-one-out analysis to ensure the robustness of the study results.

**Results:**

The results of the MR analyses indicated that hypothyroidism was associated with an increased risk of coronary heart disease (IVW: OR=2.75, 95% CI: 1.53-4.94). In mediation analyses, the proportion of HbA1c-mediated effects of hypothyroidism on coronary heart disease was 7.3% (2.2%-12.5%).

**Conclusion:**

The results of our study indicate a causal relationship between hypothyroidism and coronary heart disease. Furthermore, HbA1c partially mediated the causal effect of hypothyroidism on coronary heart disease. Consequently, intervention in this factor may reduce the risk of coronary heart disease associated with hypothyroidism.

## 1 Introduction

The thyroid gland is an endocrine organ that secretes thyroid hormones to regulate a variety of physiological and pathophysiological processes (1). Hypothyroidism is a prevalent endocrine disorder characterised by diminished levels of thyroid hormones resulting from a reduction in the synthesis or secretion of the corresponding hormones (2, 3). Cardiovascular disease represents the largest contributor to global mortality. It is anticipated that by 2025, the number of deaths due to cardiovascular disease will exceed 500,000 in men and 2.8 million in women worldwide (4). Coronary heart disease (CHD), the most prevalent form of cardiovascular disease, necessitates a high level of attention and control. Previous studies have demonstrated that the incidence of cardiovascular events is higher among patients with hypothyroidism (5), and that thyroid hormones play an important role in the normal function of the heart and blood vessels (2). Furthermore, hypothyroidism can accelerate the progression of atherosclerosis (6). Hypothyroidism exerts a profound effect on cardiovascular disease (7). Hypothyroidism is strongly associated with coronary heart disease, with possible causes including abnormal blood glucose (8), inflammatory response (9), oxidative stress (9), dyslipidaemia (10) and so on. In the clinic we found many patients with hypothyroidism combined with diabetes mellitus, so we chose diabetes mellitus related glycemic traits to be explored from the clinical reality, and finally returned to the clinic and guided the clinical practice. One study also noted that the association between these two common diseases is unlikely to be a simple coincidence (11), and another paper noted found that higher TSH levels and lower FT4 levels are associated with an increased risk of diabetes and progression from prediabetes to diabetes (12).

Furthermore, an observational study has demonstrated that hypothyroidism is a risk factor for the development of diabetes mellitus (13), and that thyroid hormones play a crucial role in glucose homeostasis and help to regulate cardiac function and the peripheral vascular system (14). These studies cannot reliably infer causality because confounders and reverse causal bias can distort the results.

Mendelian randomization is a genetic epidemiological method that employs genetic variation as an instrumental variable to investigate potential causal relationships between exposure factors and clinical disease (15). The use of genetic variants associated with risk factors as instrumental variables allows for the avoidance of bias due to confounding factors. Therefore, we employed Mendelian randomization to investigate the causal relationship between hypothyroidism and coronary heart disease, as well as to examine the relationship between glycemic traits and hypothyroidism in the context of coronary heart disease. Additionally, we sought to identify potential therapeutic strategies for coronary heart disease that do not involve the control of hypothyroidism.

## 2 Methods and materials

### 2.1 Study design

**Figure 1:**
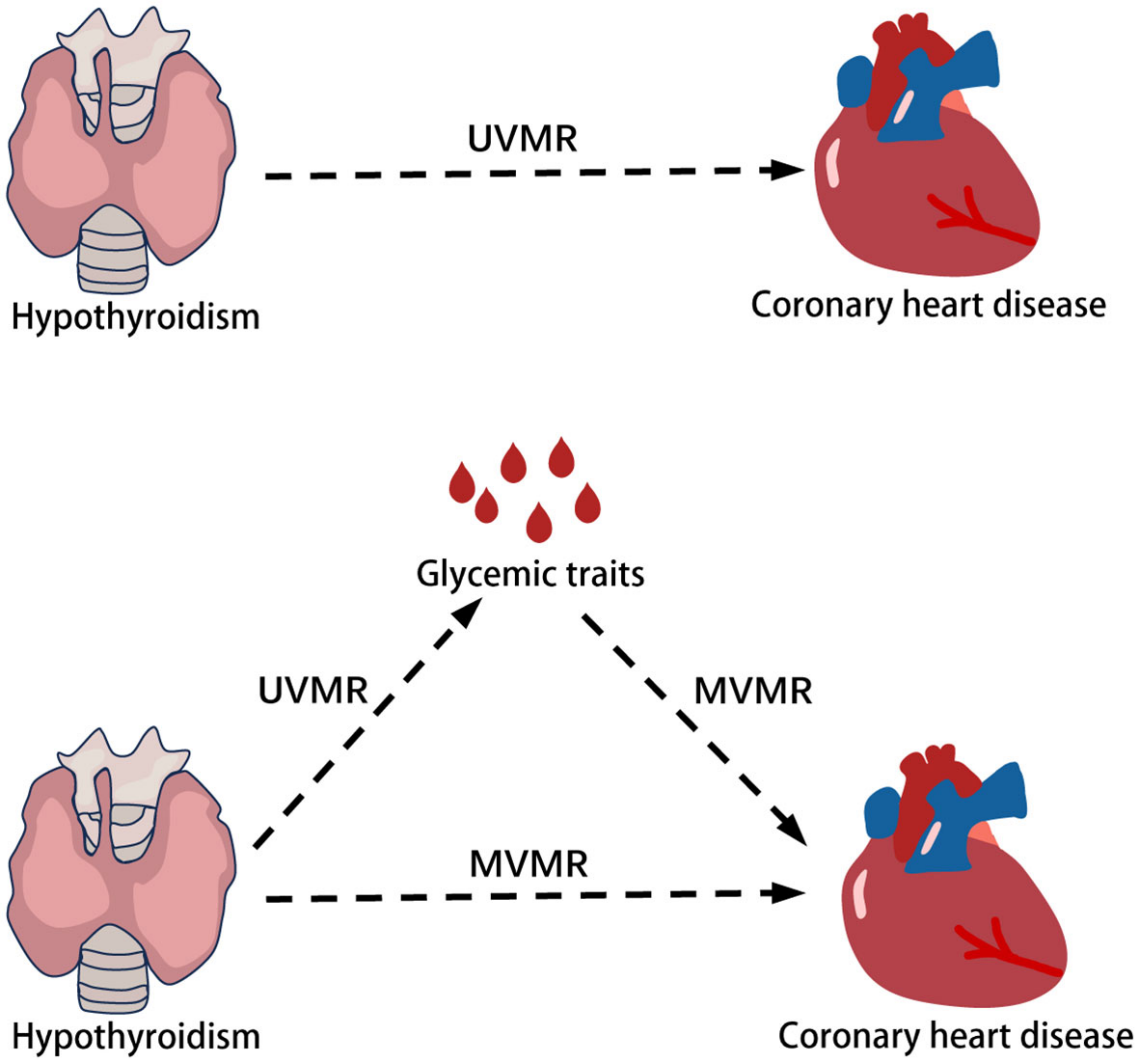
Flow chart of overall design in the present study.

**Figure 1** illustrates the initial investigation into the causal impact of hypothyroidism on coronary heart disease, employing two-sample Mendelian randomization (UVMR). The second step of the study sought to explore and quantify the potential mediating role of glycemic traits in this relationship. This was achieved through the use of multivariate Mendelian randomization (MVMR).

### 2.2 Data sources

The genetic variables were obtained from large open genome-wide association studies (GWAS) conducted on different populations. The data on hypothyroidism (ukb-b-19732, N=462,933 Europeans) and coronary heart disease (ieu-a-7, N=184,305 Europeans) were obtained from the IEU Open GWAS project database.

The data set comprises the largest GWAS to date, namely the Meta-Analyses of Glucose and Insulin-related traits Consortium (MAGIC). Including 2-hour glucose (N=63,396 Europeans), fasting glucose (N=200,622 Europeans) and fasting insulin (N=151,013 Europeans), as well as the GWAS for HbA1c, which was obtained from the UK Biobank (UKB) (N=389,889 Europeans).

The data used in this study were obtained from publicly accessible databases, and thus no ethical review was required.

### 2.3 Instrumental variable selection

In order to obtain reliable instrumental variables, the three basic assumptions of Mendelian randomization must be met (16):

- Instrumental variables are strongly correlated with exposure factors;
- Instrumental variables are not correlated with possible confounders;
- Instrumental variables are not correlated with outcome variables.

A p-value threshold of 5 × 10^-8^ was established to select for genetic variation associated with exposure. Furthermore, single nucleotide polymorphisms (SNPs) were clustered using the European LD reference panel, which removes linkage disequilibrium (LD) with an r^2^ > 0.001 over a 10,000 kb range (17). To prevent the introduction of bias due to weak instrumental variables, the F statistic was calculated, and MR analyses were considered to have strong correlation and no weak bias for instrumental variables if the F value exceeded 10 (18).

### 2.4 Statistical analysis

The causal effect of hypothyroidism on coronary heart disease was estimated using two-sample Mendelian randomization (UVMR) method. The inverse variance weighted (IVW) method (19) was employed as the primary approach for estimating the causal effect of hypothyroidism on coronary heart disease. The results are presented in terms of the ratio of ratios (OR) and 95% CI. The IVW method was considered to provide suggestive evidence of a potential association when the p-value was less than 0.05. Two-sample Mendelian randomization (UVMR) was employed to ascertain the impact of hypothyroidism on glycemic traits. The results are presented in terms of the beta coefficient and 95% confidence interval. The Bonferroni correction was employed to address the issue of multiple comparisons, with p-value thresholds considered significant for p < 0.05/4. Subsequently, we obtained estimates of the effect of the glycemic traits on moderating the effect of hypothyroidism on coronary heart disease by multivariate Mendelian randomization (MVMR) (20). The results are presented as odds ratios (OR) with 95% confidence intervals (CI). The Bonferroni-corrected p-value threshold was considered significant.

### 2.5 Mediation analysis

Firstly, the overall effect can be decomposed into an indirect effect (through mediators) and a direct effect (without mediators). In other words, the overall effect of hypothyroidism on coronary heart disease can be decomposed into two components: the direct effect of hypothyroidism on coronary heart disease and the indirect effect of hypothyroidism, which is mediated through the mediator. The mediated effect of the glycemic traits was calculated by multiplying the estimate of the effect of hypothyroidism on the glycemic traits by the estimate of the effect of the glycemic traits on coronary heart disease, respectively (21). The mediating effect was then divided by the direct effect of hypothyroidism on coronary heart disease to obtain the percentage of the mediating effect that was mediated. Additionally, 95% confidence intervals were calculated using the delta method.

### 2.6 Sensitivity analysis

The presence of pleiotropy in the data was evaluated, and the robustness of the results was assessed by means of the MR-Egger intercept test (22). If the p-value was greater than 0.05, it was concluded that there was no pleiotropy. Conversely, if the p-value was less than 0.05, it was determined that there was pleiotropy, and the results were considered to be unreliable. The heterogeneity between SNP-specific causal estimates was evaluated using the Cochran Q statistic (23). If the p-value was greater than 0.05, indicating no heterogeneity. However, if the p-value was less than 0.05, indicating heterogeneity, the random-effects model was adopted. The influence of a single SNP on the study findings was evaluated using leave-one-out analysis (24).

All of the aforementioned analyses were conducted using the R software (version 4.2.2) via the TwoSampleMR package, the Rmediation package, the Mendelian Randomization package, and the MVMR package.

## 3 Results

### 3.1 The overall impact of hypothyroidism on coronary heart disease

**Figure 2:**
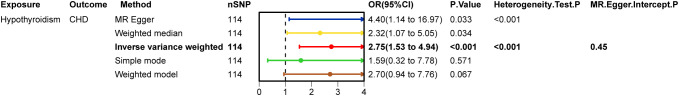
MR analysis for the causal effect of hypothyroidism on coronary heart disease.

**Figure 3:**
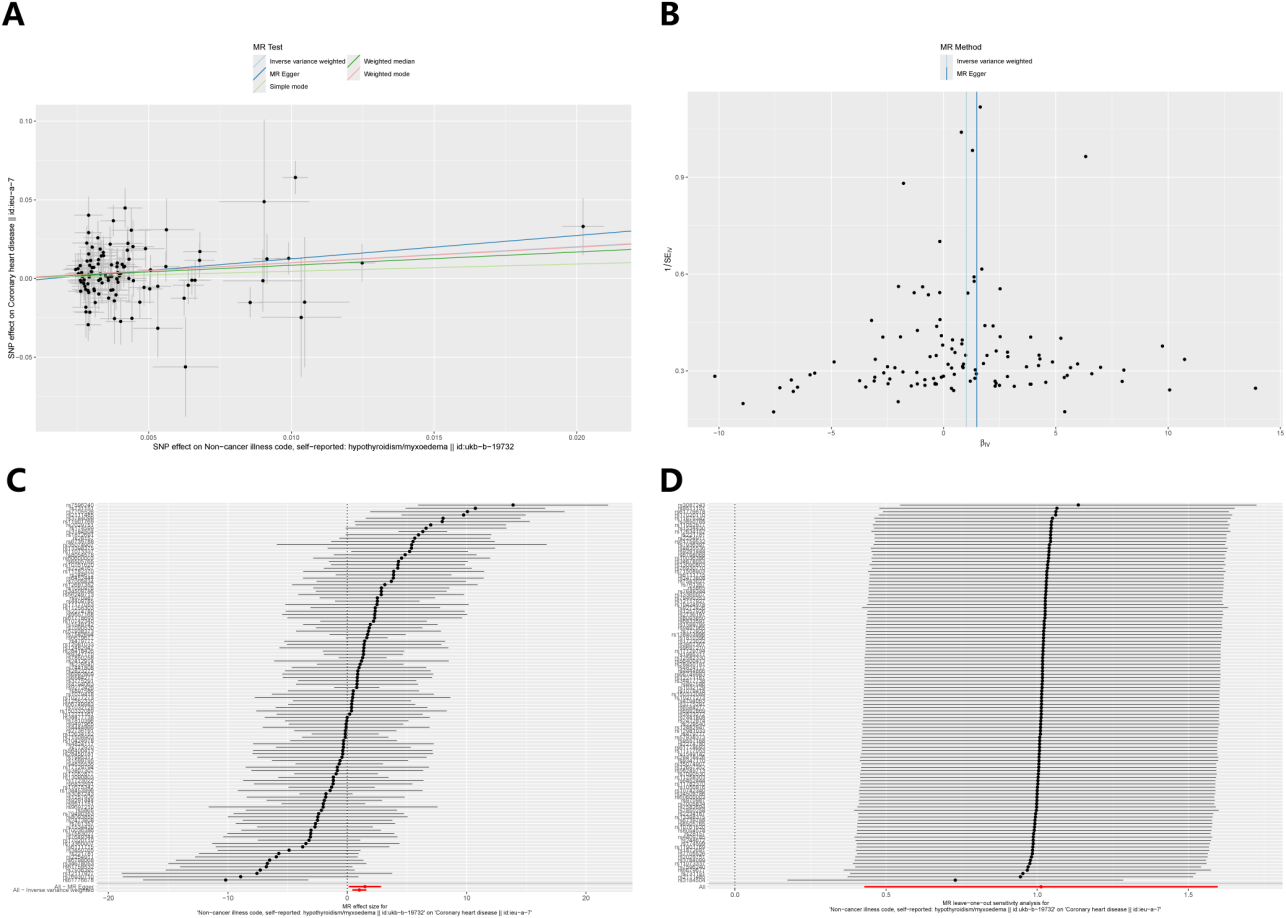
Sensitivity analysis of the causal relationship between hypothyroidism and coronary heart disease: **(A)** Scatter plots; **(B)** Funnel plots; **(C)** forest plots; **(D)** leave-one-out plots.

After excluding SNPs that did not meet the genome-wide significance criteria and linkage disequilibrium, we obtained 114 SNPs that are associated with hypothyroidism and coronary heart disease. **Figure 2** demonstrates strong evidence in favour of hypothyroidism’s causality (IVW: OR=2.75, 95% CI 1.53-4.94, p < 0.001), and the above results survived Bonferroni correction. In sensitivity analyses (**Figure 3**), MR-Egger intercept test did not detect a significant horizontal pleiotropic effect (p=0.450). Cochran’s Q test indicated the presence of heterogeneity (p<0.001), which may be attributed to a number of factors, including differences in the enrolment populations, the distinct analytical platforms employed, and the single-nucleotide polymorphisms (SNPs) under consideration. The differences in the enrollment population were attributable to differences in the genotype of the population, differences in the environment and lifestyle of the population, and differences in the sample size of the enrollment population. Due to the heterogeneity, the random-effects model in IVW was employed for data analysis. Leave-one-out analysis demonstrated that the presidential error line exhibited minimal change after the exclusion of each SNP, indicating that the findings were not influenced by a single SNP. Furthermore, the F-statistic for these SNPs was greater than 10, indicating that our results are unlikely to be biased by weak instrumentation.

### 3.2 The impact of hypothyroidism on glycemic traits

**Figure 4:**
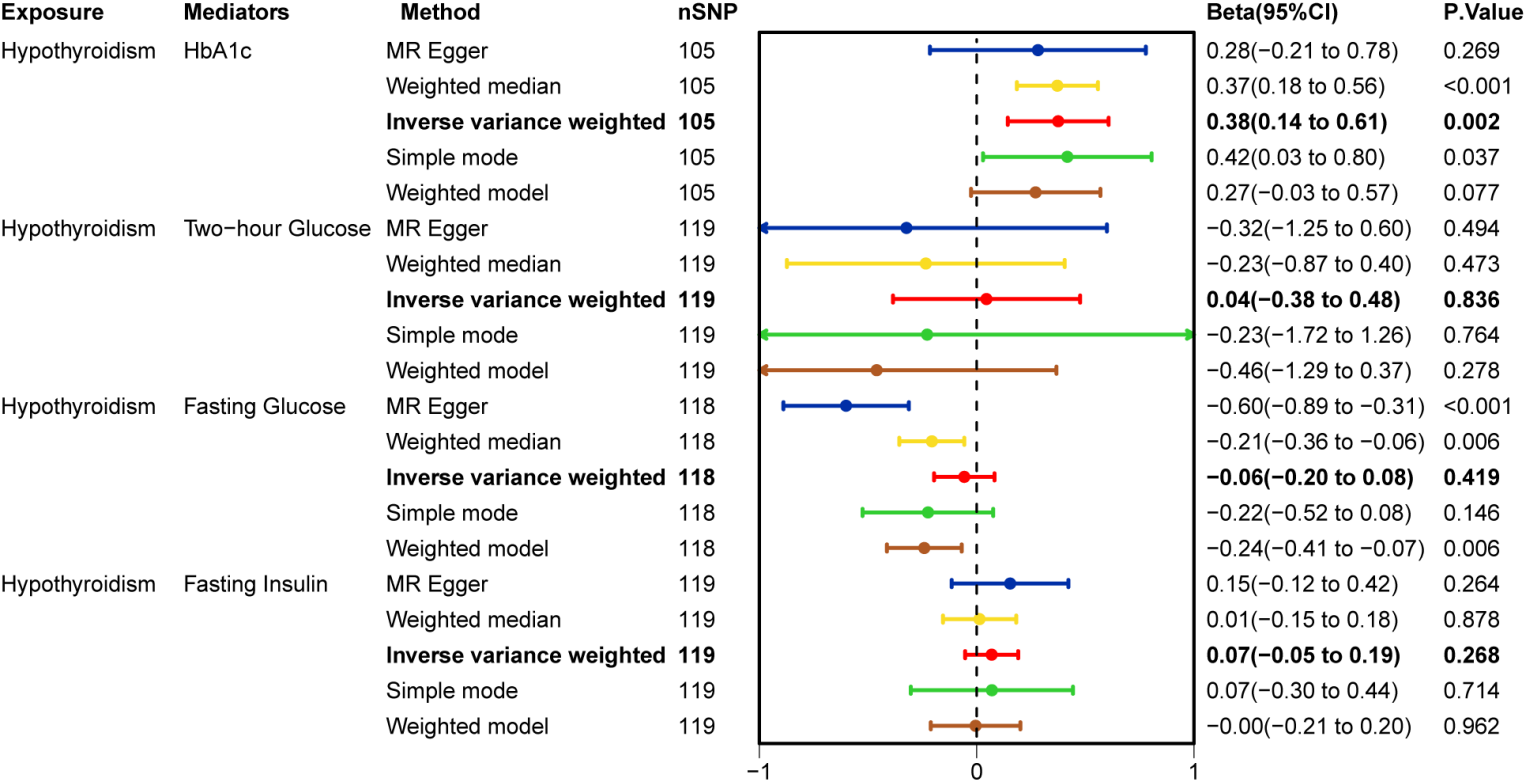
MR analysis for the causal effect of hypothyroidism on glycemic traits.

**Figure 5:**
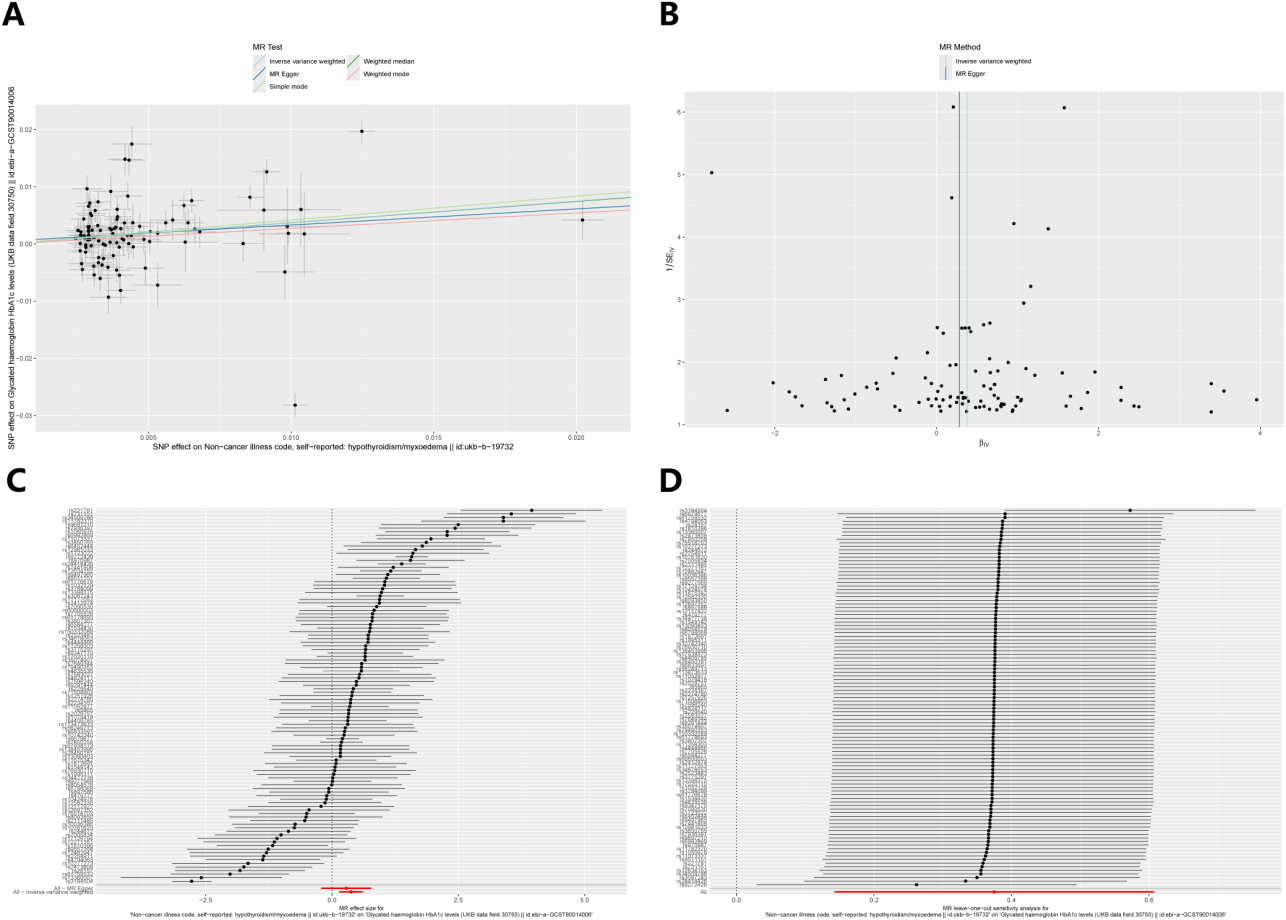
Sensitivity analysis of the causal relationship between hypothyroidism and HbA1c: **(A)** Scatter plots; **(B)** Funnel plots; **(C)** forest plots; **(D)** leave-one-out plots.

**Figure 4** illustrates the causal effect of hypothyroidism on HbA1c (IVW: β = 0.37, 95% CI: 0.14–0.61, p = 0.002). Furthermore, the aforementioned results demonstrated resilience to the Bonferroni correction. Furthermore, (**Figure 5**) MR-Egger intercept test did not detect a significant horizontal pleiotropic effect (p=0.677). Cochran’s Q test revealed the presence of heterogeneity (p<0.001), and due to the presence of heterogeneity, we chose the random-effects model in IVW for the data analysis. Leave-one-out analysis demonstrated that the presidential error line did not change much after eliminating each SNP, indicating that the findings were not affected by a single SNP. The F-statistic for these SNPs was greater than 10, indicating that our results are unlikely to be biased by weak instrumentation. The results of the study indicated that there was no causal effect of hypothyroidism on 2-hour glucose (p = 0.836), fasting glucose (p = 0.419), and fasting insulin (p = 0.268). In addition, we tested the causal effect of HbA1c on hypothyroidism and did not find a causal effect (p=0.481).

### 3.3 The effect of glycemic traits on coronary heart disease

After the exclusion of SNPs that did not meet genome-wide significance criteria and linkage disequilibrium, **Table 1** illustrates the causal effect of HbA1c on coronary heart disease after adjustment for hypothyroidism (IVW: OR=1.22, 95% CI: 1.14–1.30, p < 0.001). Furthermore, the aforementioned results were found to be statistically significant after Bonferroni correction. Furthermore, MR-Egger intercept test did not detect a significant horizontal pleiotropic effect (p=0.220). Cochran’s Q test revealed the presence of heterogeneity (p<0.001), and therefore, we chose the random effects model. The F statistic was greater than 10, suggesting that our results are unlikely to be biased by weak instrumentation.

**Table 1:** MVMR of the correlation between hypothyroidism, HbA1c and coronary heart disease.

Outcome	Variable	Method	OR	SE	P	Conditional F-statistics	MVMR heterogeneity test	MVMR directional pleiotropy test
CHD	Hypothyroidism HbA1c	MV-IVW	3.77	0.42	0.001	16.078	<0.001	0.22
	HbA1c		1.22	0.03	<0.001	115.958		
		MVMR-Egger	3.23	0.43	0.007			
			1.15	0.06	0.017			

### 3.4 The mediating effect of glycemic traits on coronary heart disease

After excluding glycemic traits that were not causally affected by hypothyroidism and those that did not have a causal effect on coronary heart disease, we employed HbA1c for mediation analysis. With regard to the causal effect of hypothyroidism on coronary heart disease, the percentages indicating HbA1c mediation were found to be 7.3% (2.2%-12.5%).

### 3.5 Summary of pleiotropy tests, heterogeneity tests and sensitivity analyses

Heterogeneity tests, multiple validity tests and sensitivity analyses were performed to assess the robustness of the MR analysis results between hypothyroidism, glycemic traits and coronary heart disease.

Hypothyroidism on coronary heart disease was first analysed (**Table 2**), MR-Egger intercept test to assess pleiotropy showed no horizontal pleiotropy (p=0.450), Cochran Q test found heterogeneity (p<0.001), random effects model in IVW was chosen for data analysis. leave-one-out method did not find that any single SNP had a large impact on the conclusions (**Figure 2**).

**Table 2:** Summary of pleiotropy tests and heterogeneity tests.

Exposure/meditor	Outcome	Pleiotropy		Heterogeneity	
		Horizontal pleiotropy(Egger intercept)	Horizontal pleiotropy(p)	Heterogeneity(Q)	Heterogeneity(p)
Hypothyroidism	CHD	-0.002	0.450	184	<0.001
Hypothyroidism	HbA1c	<0.001	0.677	626	<0.001
HbA1c	CHD	0.002	0.220	705	<0.001

As no statistical difference was found between hypothyroidism and 2-hour glucose, fasting glucose and fasting insulin. Therefore, we only analysed hypothyroidism on HbA1c (**Table 2**). MR-Egger intercept test to assess pleiotropy showed no horizontal pleiotropy (p=0.677), Cochran Q test found heterogeneity (p<0.001), random effects model in IVW was chosen for data analysis. leave-one-out method did not find that any single SNP had a large impact on the conclusions (**Figure 4**).

Finally, HbA1c and coronary heart disease were analysed after multivariate Mendelian randomisation adjusted for hypothyroidism. (**Table 2**), the MR-Egger intercept test to assess for pleiotropy showed no horizontal pleiotropy (p=0.220), the Cochran Q test found heterogeneity (p<0.001), and the random effects model in IVW was selected for data analysis.

## 4 Discussion

Mendelian randomization, which employs genetic variation as an instrumental variable to infer causality (16, 25, 26), has emerged as a reliable statistical method. As genetic variation is present at birth and is not influenced by environmental factors, it reduces the possibility of confounding variables and reverse causation (27). The use of a large genetic database in Mendelian randomization studies allows for more precise results. In this context, the aim is to explore the relationship between hypothyroidism and coronary heart disease and to investigate the role of glycemic traits in this relationship. Two-sample, multivariate Mendelian randomization and mediation analyses were performed using relevant resources from the GWAS database. The results indicated that hypothyroidism is a risk factor for the incidence of coronary heart disease (IVW: OR=2.75, 95% CI: 1.53-4.94). Furthermore, the proportionate effect of HbA1c-mediated hypothyroidism on coronary heart disease was 7.3% (2.2%-12.5%). To further validate the robustness and reliability of our findings, we verified that our results are robust and reliable using MR-Egger intercept test, Cochran’s Q test, and leave-one-out analysis. MR-Egger intercept test did not detect a significant horizontal pleiotropic effect. Cochran’s Q test revealed the presence of heterogeneity, we chose the random-effects model in IVW for the data analysis. Leave-one-out analysis, which did not identify a single instrumental variable as a significant contributor to the outcome variable.

Hypothyroidism is defined as a condition in which there is a reduction in the secretion or production of thyroid hormones, as evidenced by elevated TSH levels. The present study aimed to elucidate the multiple pathways by which hypothyroidism leads to coronary heart disease. Our study was based on clinical findings. Therefore, only glycaemic factors associated with diabetes were examined. we found that HbA1c plays a mediating role in the process of coronary heart disease due to hypothyroidism. There are also other possible mechanisms by which hypothyroidism causes coronary heart disease that we have identified in the literature of other investigators. For example, inflammatory response (9), oxidative stress (9), dyslipidaemia (10), etc. Interestingly, we found that diabetes also plays a role in these factors, which could be the reason or mechanism for the mediating role of diabetes and related glycaemic factors. a) Inflammatory response: Hypothyroidism can lead to an inflammatory response (9), and hyperglycaemia itself can increase the inflammatory response (28). This leads to activation of inflammatory cells and release of inflammatory factors that can damage blood vessel walls and accelerate the process of atherosclerosis (29). b) Oxidative reactions: Hypothyroidism can lead to oxidative stress (9), and hyperglycaemia promotes the production of reactive oxygen species (ROS), which increases oxidative stress.ROS oxidise low-density lipoproteins (LDL), and oxidised LDL is more readily taken up by macrophages, which form foam cells, a key step in the formation of atherosclerotic plaques (28, 30). c) Dyslipidaemia: Some studies have suggested that patients with hypothyroidism may also have dyslipidaemia, such as changes in high cholesterol and triglyceride levels (31, 32). These are independent risk factors for coronary heart disease. Diabetic status may exacerbate this dyslipidaemia (33). However, other analyses have concluded that hypothyroidism is not associated with lipids (34), and this is controversial.

The present study has identified a causal relationship between hypothyroidism and coronary heart disease, and has also demonstrated the role of HbA1c in this relationship. Taking into account the existing literature, we found that HbA1c is closely related to the risk of developing coronary heart disease (35–37). In people with type 2 diabetes, an unstable HbA1c trajectory is associated with a greater risk of microvascular events and mortality (38), with an approximately 13% increased risk of cardiovascular events for every 1% increase in HbA1c (39). Furthermore, controlling HbA1c levels to <7.0% significantly slows the progression of coronary artery calcification, thereby reducing the incidence of cardiovascular disease in patients (40). It is therefore reasonable to assume that interventions on HbA1c in hypothyroid patients may also reduce the risk of coronary heart disease. This plays a very important role in guiding clinical work. Consequently, it is recommended that patients with hypothyroidism be advised to detect, diagnose and treat the condition promptly, in order to prevent the development of coronary heart disease. In patients with hypothyroidism who have abnormal glycemic traits, prompt glycemic control is of even greater importance. When treating patients with hypothyroidism, we should control TSH levels. In the absence of large randomised controlled trials, the best evidence suggests that treatment for hypothyroidism should be initiated at TSH ≥ 10 mIU/L; there is no precise threshold for specific control targets, but it is clear that patients with cardiovascular disease or at significantly higher risk of hypothyroidism may benefit from early treatment (41). For patients with hypothyroidism who also have diabetes, HbA1c levels below 7.0% are recommended to reduce cardiovascular events (40).

Nevertheless, it should be noted that this study is not without limitations. Firstly, the use of the European population sample avoids the potential for bias due to ethnic and geographical stratification. However, this approach also limits the possibility of generalising the findings of our study to the wider population. To increase the breadth of our findings, we drew on relevant studies from different regions and a large body of literature. For example, a comprehensive analysis of 55 cohort studies in North America, involving almost 1.9 million participants, found that patients with hypothyroidism had a slightly higher risk of cardiovascular events (42). In a prospective cohort study in Korea, high thyrotropin levels were associated with a higher risk of death and new cardiovascular disease, especially in subjects at high risk of cardiovascular disease (43). Hypothyroidism was also associated with increased cardiovascular mortality in a large population-based study in Denmark (44). A Norwegian study with 12 years of follow-up concluded that high TSH levels were associated with increased mortality from coronary heart disease (45). This literature suggests that the association of certain genetic variants with disease risk is fairly consistent across populations. Future studies should include more samples from different populations to assess the prevalence and specificity of genetic effects in different populations and to improve the general applicability of the findings. Secondly, the use of pooled GWAS data precluded the stratification of the data according to factors such as age and gender. Thirdly, both hypothyroidism and diabetes are common diseases in daily life. For example, the likelihood of developing diabetes varies according to age, dietary habits and whether or not you exercise; hypothyroidism is similarly linked to dietary habits (whether or not you have an iodine deficiency) and whether or not you have immune thyroiditis, which we have not been able to study in more detail. Furthermore, our study indicates a causal relationship between hypothyroidism and coronary heart disease, with a small portion of the effect mediated by HbA1c. However, the majority of the effect of hypothyroidism on coronary heart disease remains unknown. This also indicates the existence of a complex relationship between hypothyroidism and coronary heart disease. Further study of other risk factors as potential mediators is required.

In a follow-up study, we plan to do a retrospective cohort study: select medical records from a certain period in the past, make sure to include both patients and non-patients with hypothyroidism, look at the medical records to see if they had coronary heart disease, and obtain HbA1c data from laboratory data. The association between hypothyroidism and coronary heart disease was assessed using Cox proportional risk regression modelling. The mediating role of HbA1c was assessed using mediation analysis. In addition, appropriate basic experiments will be carried out: animal model studies and analysis of biomarkers (e.g. inflammatory factors, lipid metabolism) will be used to investigate how HbA1c affects relevant biological pathways. This will help us understand the specific role of HbA1c in the relationship between hypothyroidism and coronary heart disease. These steps will allow a more efficient transition from mechanism research to specific clinical applications aimed at validating and exploiting HbA1c as a potential therapeutic target to reduce the risk of coronary heart disease in patients with hypothyroidism.

## 5 Conclusion

There is a causal relationship between hypothyroidism and coronary heart disease. Furthermore, hypothyroidism increases a patient’s risk of coronary heart disease. HbA1c mediates part of the causal effect of hypothyroidism on coronary heart disease. Therefore, intervention in this factor may reduce the risk of hypothyroidism on coronary heart disease.

## Data Availability

The original contributions presented in the study are included in the article/Supplementary Material. Further inquiries can be directed to the corresponding author.
